# Right heart failure after left ventricular assist device implantation: latest insights and knowledge gaps on mechanism and prediction

**DOI:** 10.3389/fcvm.2025.1586389

**Published:** 2025-05-22

**Authors:** Hideaki Nonaka, Lawrence Y. Lu, Nchafatso G. Obonyo, Jacky Y. Suen, David C. McGiffin, Jonathon P. Fanning, John F. Fraser

**Affiliations:** ^1^ Critical Care Research Group, The Prince Charles Hospital, Brisbane, QLD, Australia; ^2^ Institute for Molecular Bioscience, University of Queensland, Brisbane, QLD, Australia; ^3^ Division of Surgery, Princess Alexandra Hospital, Brisbane, QLD, Australia; ^4^ Wellcome Trust Centre for Global Health Research, Imperial College London, London, United Kingdom; ^5^ Initiative to Develop African Research Leaders/KEMRI-Wellcome Trust Research Programme, Kilifi, Kenya; ^6^ School of Pharmacy and Medical Sciences, Griffith University, Southport, QLD, Australia; ^7^ Cardiothoracic Surgery and Transplantation, The Alfred Hospital, Melbourne, VIC, Australia; ^8^ Department of Surgery, Monash University, Melbourne, VIC, Australia; ^9^ Anesthesia & Perfusion Services, The Prince Charles Hospital, Brisbane, QLD, Australia; ^10^ Intensive Care Unit, St Andrews War Memorial Hospital, Brisbane, QLD, Australia

**Keywords:** LVAD, left ventricular twist, pressure-volume loop, PV loop, RHF, right ventricle, RVF, score

## Abstract

Heart failure is a global health concern, with many patients being unresponsive to medical therapies. In end-stage disease, left ventricular assist devices (LVADs) offer an alternative to transplantation, yet their clinical course remains unfavorable, with up to one in four patients dying within a year. Although LVAD implantation aims to alleviate left-sided congestion and reduce right ventricular burden, a significant proportion of patients develop RHF, which is a major driver of morbidity and mortality. The underlying mechanisms leading to RHF remain a subject of debate, with no definitive conclusions reached. Due to the heterogeneity of heart failure pathophysiology, clinical data varies, and the translation of preclinical findings into effective bedside management remains challenging. These factors collectively hinder the precise characterization of RHF mechanisms, with some proposed explanations remaining speculative. Assessing the risk of RHF development based on pathophysiological insights is essential. However, predicting the progression of RHF following LVAD implantation remains difficult due to complex hemodynamic interactions and the lack of established guidelines, often leading to missed opportunities for timely right ventricular (RV) support device implantation. To reduce the incidence of RHF, this review aims to provide insights into RV failure mechanisms and propose a refined predictive approach. Although data in this field is rapidly evolving, explanations and assessment methods have not been significantly updated. This paper consolidates recent findings, presents updated perspectives, and identifies remaining gaps in knowledge.

## 1 Introduction

Heart failure (HF) affects more than 65 million worldwide, with many patients being unresponsive to medical therapies (1). For those with end-stage HF, mechanical circulatory support (MCS), in the form of left ventricular assist devices (LVADs), has emerged as a bridge to transplantation, to a decision, or to myocardial recovery, as well as destination therapy in patients in whom cardiac transplantation is contraindicated (2).

Despite advances in LVAD indications, the incidence of right heart failure (RHF) after LVAD implantation remains a major concern, where the dysfunctional right ventricle (RV) undermines hemodynamic stability and is a driver of morbidity and mortality (3, 4). Previous studies report that 10%–35% of patients experience RHF within one month following LVAD placement (5–8) and the in-hospital mortality rate of LVAD recipients requiring right ventricular assist device (RVAD) was up to 50%, while patients undergoing planned biventricular assist device (BiVAD) placement face a mortality rate of 30% (6).

Considering that LVADs relieve congestion in the left-side heart and, consequently, the right side, the underlying mechanism of RHF is challenging to fully elucidate. Several contributing factors have been discussed in previous reviews, including excessive LVAD suction disrupting ventricular interdependence and increased LVAD flow elevating RV preload, yet adequate evidence supporting some of these phenomena is lacking, with speculative components included. Additionally, it remains unclear which mechanisms are well supported by firm evidence and which remain hypothetical (9). Most of the current evidence relies on load-dependent functional parameters, further raising concerns about their reliability in LVAD patients, where loading conditions fluctuate dramatically (10–12).

Additionally, planned BiVAD implantation potentially improves the outcomes as mentioned previously (6), yet the definitive indication for simultaneous LV and RV support device implantation is unclear. Although numerous factors have been proposed as predictors for RHF after LVAD implantation, their reproducibility remains inconsistent.

To advance our understanding of the mechanism and possibly improve the prediction of post-LVAD RHF, this review aims to: (i) examine the recently proposed pathophysiological mechanistic evidence from the human pressure-volume (PV) loops to capture load-independent functional parameters such as RV end-systolic elastance (Ees) and end-diastolic pressure volume relation (EDPVR); (ii) evaluate existing functional assessment approaches and risk scores to predict RHF development, and consolidate the key factors; and, (iii) clarify the current knowledge gaps and future research directions. To capture the latest insights, we conducted an extensive literature search in PubMed MEDLINE, Scopus, Embase, and ClinicalTrials.gov using controlled keywords, primarily “LVAD” and “RHF.”

**Figure 1:**
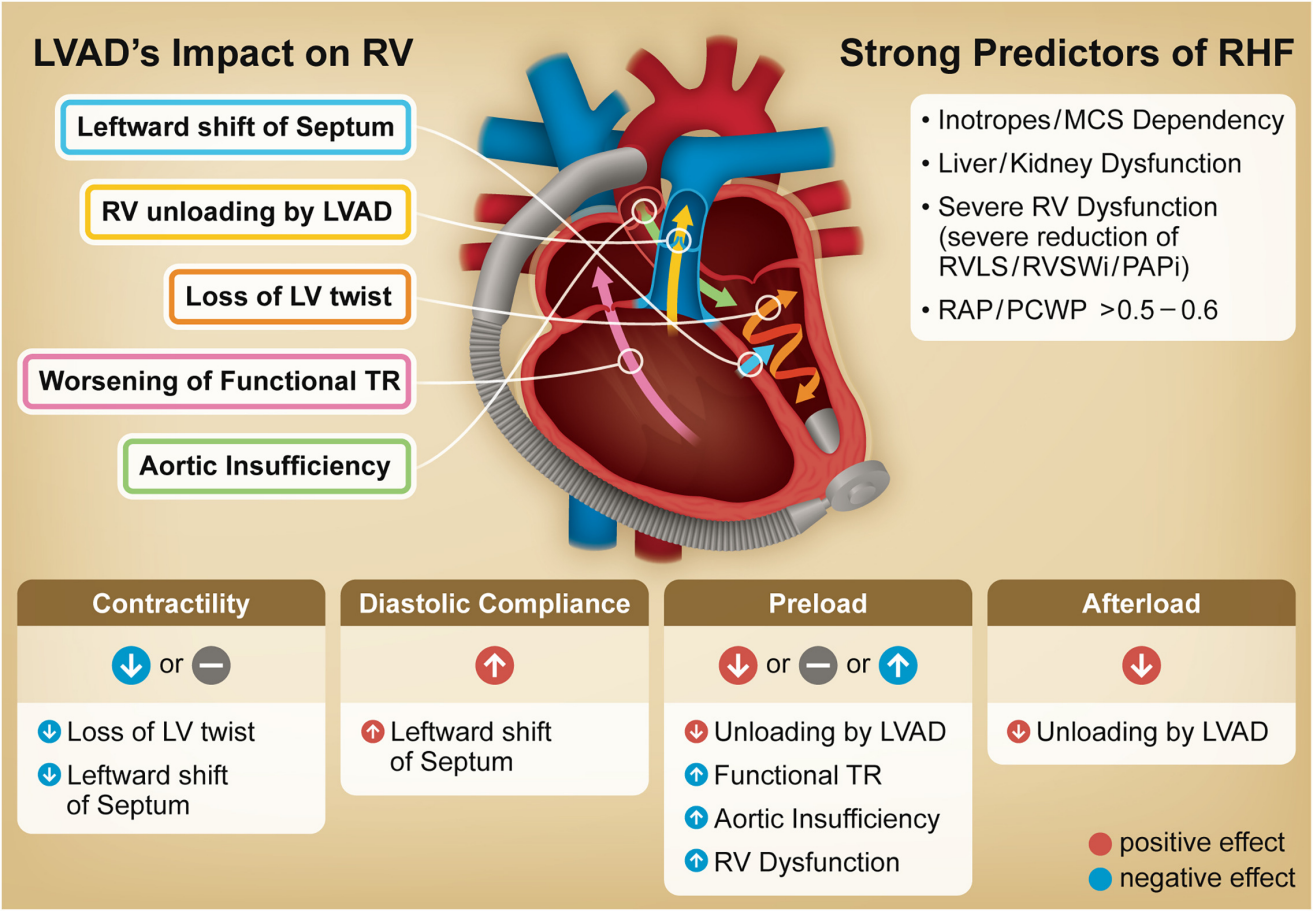
Graphical abstract. Illustration outlines the primary impact of an LVAD on the RV, including underlying mechanisms driving post-LVAD RHF. The table at the bottom summarizes changes in RV function and loading conditions following LVAD implantation. The column on the right identifies strong predictors for RHF including non-cardiac factors, all of which are incorporated into at least two representative risk scores and have been identified as significant predictors in large scale multivariable analyses. LVAD, left ventricular assist device; TR, tricuspid regurgitation; RV, right ventricle; RHF, right heart failure; MCS, mechanical circulatory support; RV LS, right ventricular longitudinal strain; RVSWi, right ventricular stroke work index; PAPi, pulmonary artery pulsatility index; RAP, right atrial pressure; PCWP, pulmonary capillary wedge pressure.

Figure 1 illustrates the principal concepts discussed in this review. The goal of this review is to assist clinicians in optimizing LVAD management and guiding research, ultimately to improve patient outcomes.

## 2 RHF definition

The Interagency Registry for Mechanically Assisted Circulatory Support (INTERMACS) and the updated Mechanical Circulatory Support—Academic Research Consortium (MCS–ARC) definitions of RHF have been widely accepted (13, 14). In the most recent consensus statement from 2020, RHF is classified into three categories based on the timing of symptom onset (Table 1) (14):

1. Early acute RHF,
  - requiring concomitant implantation of a temporary VAD or
  - requiring RVAD with an LVAD implantation
2. Early post-implantation RHF,
  - requiring a RVAD <30 days or
  - failing to wean from inotropes, vasopressors or nitric oxide within 14 days, or
  - death attributable to RHF <14 days from an LVAD implantation
3. Late RHF,
  - requiring a RVAD more than 30 days or
  - requiring hospitalization for RHF >30 days after an LVAD implantation

**Table 1:** MCS-ARC definition of right heart failure.

RHF type	Criteria for diagnosis
Early Acute RHF	• Need for implantation of a temporary or durable RVAD (including ECMO) concomitant with LVAD implantation (i.e., the RVAD is implanted before the patient leaves the operating room).
Early post-implant RHF	• Need for implantation of a temporary or durable RVAD (including ECMO) within 30 days following LVAD implantation (for any duration of time); OR • Failure to wean from inotropes or vasopressors, or inhaled nitric oxide within 14 days following LVAD implantation; OR • Need to (re)initiate this support within 30 days of LVAD implantation for a duration of at least 14 days; OR • Death occurring within 14 days of LVAD implantation in patients who did not receive an RVAD but remained on inotropes or vasopressors at the time of death and met the diagnostic criteria for RHF (at least 2 clinical findings or 1 manifestation listed below. • Primary diagnosis of RHF (must have ≥2 of the clinical findings OR ≥1 manifestation): o Clinical Findings: ▪ Ascites ▪ Functionally limiting peripheral edema (at least moderate level of swelling in the extremities) ▪ Elevated estimated JVP (at least halfway up the neck in an upright patient) ▪ Elevated measured CVP or RA pressure (≥16 mm Hg) o Manifestations: ▪ Renal failure: serum creatinine >2 × baseline ▪ Liver injury: ≥2 × upper limit normal in AST/ALT, or total bilirubin >2.0 mg/dl ▪ S_V_O_2_ < 50% ▪ Cardiac index <2.2 L/min/m^2^ ▪ Reduction in pump flow of >30% from the previous baseline (in the absence of mechanical causes such as tamponade or ▪ tension pneumothorax) ▪ Elevated lactate >3.0 mmol/L. · · Pediatric Adaptation The above criteria may be modified for pediatric patients. Primary diagnosis of RHF requires ≥2 of the clinical findings OR ≥1 manifestation o Clinical Findings: ▪ Ascites ▪ Significant peripheral edema (at least moderate level of swelling in the extremities) ▪ Elevated JVP (visible in an upright patient) or hepatomegaly (3 + cm below costal margin) ▪ Elevated CVP or RA pressure: - Age 10–18 years: CV*P* >14 mm Hg - Age 5–10 years: CVP >12 mm Hg - Age <5 years: CVP >10 mm Hg o Manifestations: ▪ Renal failure: serum creatinine ≥1.5 × above baseline. ▪ Liver injury with an elevation of AST, ALT or total bilirubin ≥2 × upper normal. ▪ Decrease in pump flow ≥30% from a recent baseline in the absence of tamponade. ▪ We need to decrease the pump rate ≥20% or more from a recent baseline owing to the poor filling of LVAD in a pulsatile system. ▪ Cardiac Index <2.2 L/min/m^2^
Late RHF	• Need for implantation of an RVAD (including ECMO) ≥30 days after LVAD implantation. This may occur during the index hospitalization for LVAD placement or any subsequent readmission, OR • Hospitalization ≥30 days post-implant requiring intravenous diuretics or inotropic support for ≥72 h and associated with RHF by criteria below: o Diagnosis of RHF (must have ≥2 clinical findings OR ≥1 manifestation: ▪ Clinical Findings ▪ Ascites ▪ Functionally limiting peripheral edema (>2+). ▪ Elevated estimated JVP at least halfway up the neck in an upright patient. ▪ Elevated measured CVP (>16 mm Hg). o Manifestations: ▪ Renal failure: serum creatinine >2 × baseline value ▪ Liver injury: ≥2 × upper limit normal in AST/ALT, or total bilirubin >2.0 mg/dl ▪ Reduction in pump flow of >30% from the previous baseline (in the absence of tamponade) ▪ S_V_O_2_ < 50% ▪ Cardiac index <2.2 L/min/m^2^ ▪ Elevated lactate >3.0 mmol/L • Pediatric Adaptation o Requirement for intravenous diuretics or inotropic support of ≥72 h due to new onset right heart failure (i.e., not present continuously since implantation, with a period of ≥7 consecutive days off intravenous support) ▪ Diagnosis of RHF requires ≥2 of the following clinical findings, or ≥1 manifestations (as above, adjusting for pediatric CVP thresholds and definitions of ascites, edema, and hepatomegaly)

Above definition of RHF is extracted from the MCS-ARC consensus statement (14). RHF, right heart failure; LVAD, left ventricular assist device; RVAD, right ventricular assist device; ECMO, extracorporeal membrane oxygenator; AST, aspartate aminotransferase; ALT, alanine aminotransferase; SvO_2_, venous oxygen saturation; JVP, jugular venous pressure; CVP, central venous pressure; RA, right atrium.

In addition to these categories, the diagnostic criteria also include multiple clinical indicators, such as hemodynamic parameters (elevated central venous pressure, reduced cardiac index, and reduced venous oxygen saturation), right heart failure symptoms (e.g., edema, ascites), and end-organ dysfunction (e.g., kidney dysfunction, liver failure) (14). Furthermore, the statement also recommends classifying RHF incidence into three categories based on its associations: (1) patient-related (e.g., valvular heart disease, pulmonary disease, cardiorenal syndrome); (2) management-related (e.g., surgical procedures, inotrope withdrawal, volume overload); and (3) device-related (e.g., pump malfunction, outflow graft compromise). Early RHF occurs most frequently. In a recent study using the STS INTERMACS database, the prevalence of *de novo* RHF at one month (i.e., early RHF) was as high as 24% (8).

**Figure 2:**
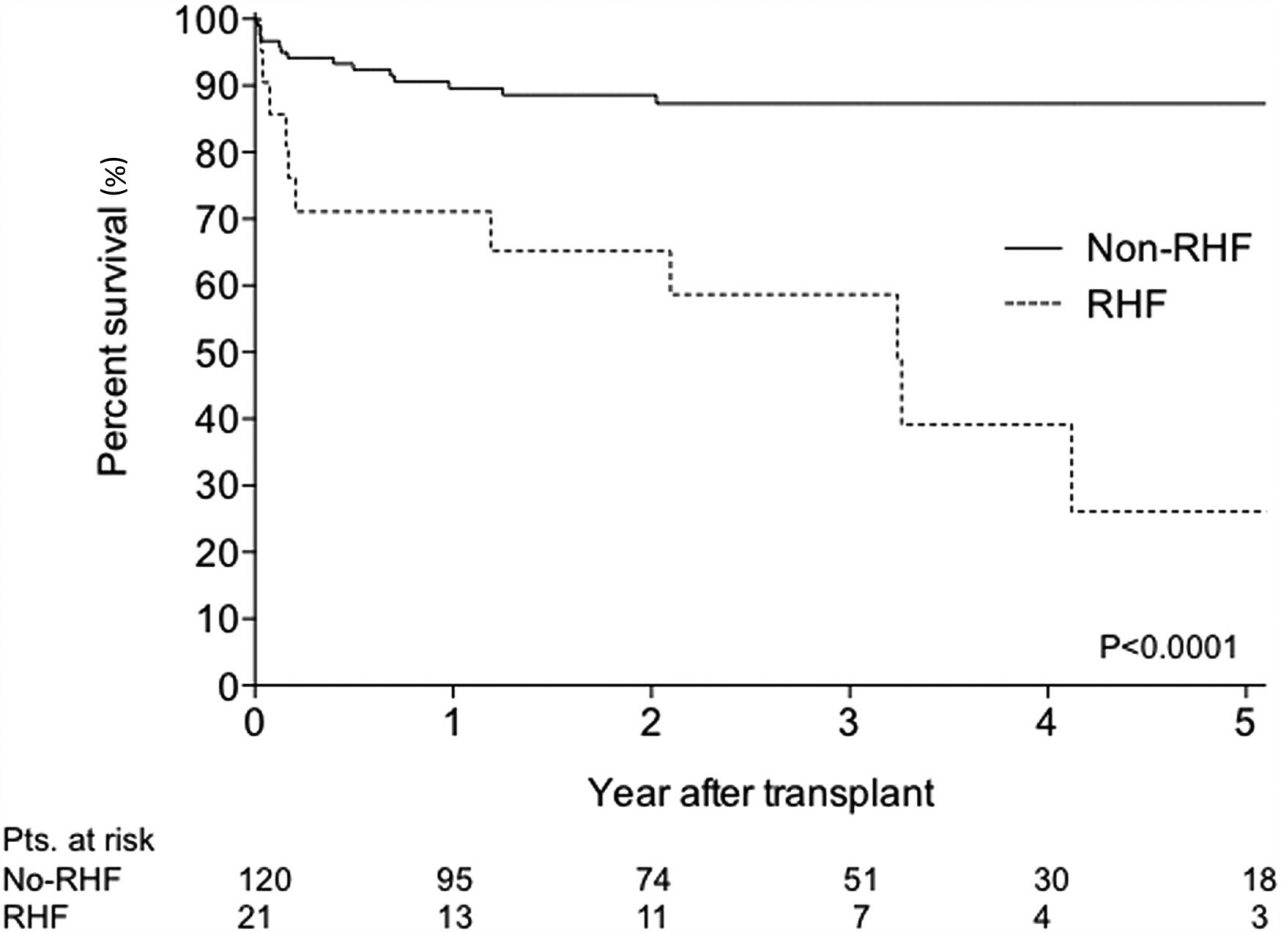
Comparison of survival rate between patients with and without right heart failure five-year kaplan-meier curve between patients with and without RHF.. RHF was defined as rehospitalization and medical treatment because of recurrent RHF or patients who required continuous inotropic support because of persistent RHF >4 weeks after implantation. The survival curve gap between the two groups widens year by year. Reproduced with permission from “Comparison of survival: non-RHF group versus RHF group. RHF, right heart failure” by Koji Takeda, Hiroo Takayama, Paolo C. Colombo, Ulrich P. Jorde, Melana Yuzefpolskaya, Shinichi Fukuhara, Donna M. Mancini and Yoshifumi Naka, licensed under CC-BY-NC-ND.

Although the overall prevalence of late RHF remains stable and less than early RHF at a rate of 8%–10% over their three-year surveillance, it cannot be underestimated (8). This is because late RHF is also associated with the least favorable outcome (8, 15). Takeda et al. demonstrated the prognostic significance of late RHF after continuous-flow LVAD implantation, with the survival of patients with RHF progressively worsening compared to that of patients without RHF (Figure 2) (16).

This definition is widely adopted in most studies and is therefore used in this review.

## 3 Mechanisms of RHF

**Figure 3:**
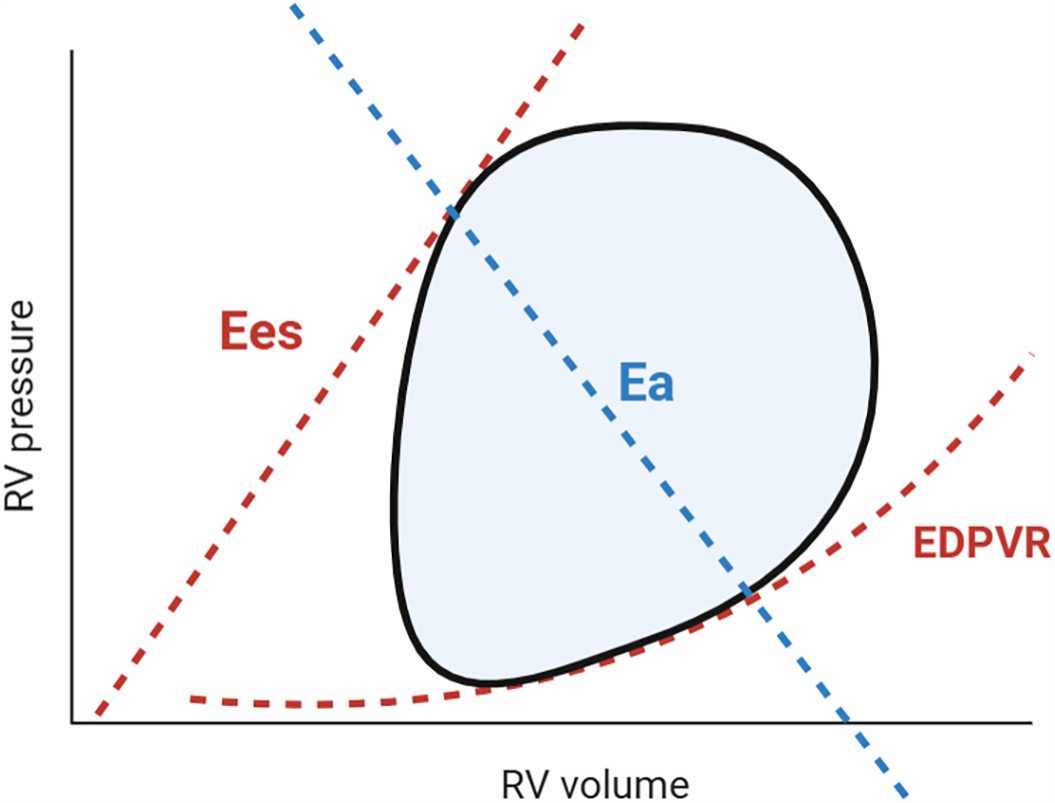
Pressure-Volume loop and right ventricular and pulmonary artery coupling. The steady-state RV PV loop (black line) illustrates the relationship between pressure and volume during a cardiac cycle. The enclosed area by the PV loop represents RV stroke work. By reducing preload (e.g., via inferior vena cava occlusion or the Valsalva maneuver), multiple PV loops can be generated, from which Ees and EDPVR can be derived. Effective pulmonary arterial elastance (Ea; blue) is a determinant of RV afterload. The RV Ees/Ea ratio describes RV-PA coupling, assessing contractility relative to load. An optimal ratio (1.5–2.0) ensures cardiac efficiency, whereas uncoupling (0.6–1.0) signifies RV decompensation (17, 20). RV, right ventricle; PV, pressure volume; Ees, end-systolic elastance; EDPVR, end-diastolic pressure volume relationship; Ea, effective arterial elastance.

This section outlines the impact of implantation of an LVAD on the RV and discusses the potential mechanisms driving RHF. In the later part of the section, PV loop data from clinical studies are summarized, as it accurately reflects afterload and preload on RV and provide load-independent functional parameters such as Ees and EDPVR (Figure 3). Moreover, PV loops also provide pulmonary artery (PA) effective arterial elastance (Ea) and the RV Ees/Ea ratio, commonly referred to as “RV-PA coupling,” which serve as valuable indices of RV contractility in relation to the afterload imposed by the PA (17). These approaches will contribute to a more comprehensive understanding of RHF mechanisms.

A fundamental understanding of interventricular interaction is essential to grasp this section, as it plays a critical role in the pathophysiology of post-LVAD RHF. The Torrent-Guasp helical model describes the entire heart as composed of two interconnected loops of the myocardium (18, 19). One loop has muscle fibers wrapping around both ventricles in parallel with a short axis at their basement, and the other loop has fibers spiraling around the left ventricle (LV), including the interventricular septum. The RV is constructed from these two fiber systems, with the free wall formed by the first (wrapping) myocardial loop and the septal wall formed by the second (helical) structure. This structure is crucial because the “helical” or “twisting” motion of the secondary loop significantly contributes to the RV systolic function. This LV “twisting” contributes to longitudinal motion of RV, while wrapping myocardium primarily facilitates “transverse (radial)” movement of RV free wall (20, 21).

According to studies in a canine model by Hoffman and colleagues, where the RV free wall was replaced with a xenograft pericardial patch, LV motion accounted for 24%–35% of RV stroke work (SW) (22). Thus, RV function can be significantly influenced by the LV through the septal wall, which is key factor in the occurrence of RHF after LVAD implantation.

### 3.1 LVAD-specific effects on RV

The following mechanisms can act individually or in combination: (1) Loss of LV twist, (2) Leftward shift of the interventricular septum, (3) Changes in RV Loading Conditions Due to LVAD, (4) Worsening of functional tricuspid regurgitation (TR), (5) Aortic insufficiency (AI). Notably, these mechanisms remain incompletely characterized in the literature, as studying the mechanisms of RHF in patients is challenging presumably due to the heterogeneity of LVAD candidates' pathophysiology and the lack of accurate human data.

#### 3.1.1 Loss of LV twist

##### - Decrease in RV contractility

In LVAD patients, LV twisting motion is primarily impaired due to two factors: (1) loss of constraint following pericardiotomy, and (2) constraining of the LV apex and septum.

The relationship between pericardial constraint and LV twist has been frequently discussed in the past. A preclinical study demonstrated a decline in LV twist, measured by LV speckle tracking, following pericardiotomy—due to the loss of pericardial constraint, suggesting that this constraint plays a significant role in LV twist. This study was specifically designed to investigate the role of the pericardium and further revealed that the decline in LV twist was restored after pericardial closure (23). Additionally, impaired longitudinal motion of the RV after pericardiotomy has been reported in clinical settings including mitral valve surgery, heart transplantation and coronary artery bypass graft (CABG) surgery, whereas other thoracic surgeries, such as lung transplantation, did not demonstrate a similar effect. Given that LV twist plays a significant role in the longitudinal movement of the RV, RHF after LVAD implantation may be attributable to the loss of LV twist. Long-term follow-up studies have reported that this reduction in longitudinal motion persists for more than one month even after chest closure (23–26). Clinical data linking the loss of LV twist to pericardiotomy specifically in LVAD implantation remain sparse, highlighting the need for further data accumulation to support this explanation.

Additionally, the LVAD replacement to the LV apex can also constrain the LV apex and septum, potentially impairing LV twisting movement (Figure 1). This deformation is considered to negatively affect RV outflow although clinical research on this impact remain limited (20). However, data directly supporting this mechanism also remain scarce, making this impact somewhat speculative. More detailed data on LV twist in LVAD patients are needed.

#### 3.1.2 Leftward shift of interventricular septum

##### - Decrease in RV contractility - Improvement in RV diastolic compliance

In an LVAD configuration, suction within the LV displaces the interventricular septum leftward, altering its geometry, reducing septal motion, and consequently diminishing RV contractility, while simultaneously improving RV diastolic compliance (9).

A decline in RV contractility by leftward shift of the interventricular septum is the subject of debate (27), and preclinical studies support this expectation (28, 29). Since the interventricular septum plays a pivotal role in RV systolic function, as previously mentioned, excessive LVAD suction can impair septal wall motion, thereby compromising RV function. A study on PV loops and septal strain for RV contractility assessment in LVAD patients found that alterations in RV Ees during ramp test were significantly correlated with RV septal strain (*r* = 0.78, *p* = 0.02) but not with free wall strain (*r* = 0.45, *p* = 0.26), highlighting the critical role of the interventricular septum in RV contractility (30). Impaired septal movement can be compensated by the RV free wall; however, this mechanism fails in patients with a high afterload. In this context, contractile impairment significantly affects RV SV, resulting in RV-PA uncoupling (20).

In contrast to contractility, leftward septal deformation can enhance RV compliance. Left-sided suction allows the RV to expand further, even under low end-diastolic pressure, thereby increasing diastolic compliance, a mechanism that will be discussed later in the PV loop part (3–2. PV loop in patients with LVAD). This effect is beneficial to the RV, and does not directly contribute to RHF (31).

#### 3.1.3 Changes in RV loading conditions due to LVAD

##### - Decrease(/Increase) in RV afterload - Decrease/Increase in RV preload

To avoid confusion, this paper defines afterload/preload as end-systolic pressure (ESP) and end-diastolic pressure (EDP), respectively as per Guyton and Hall Textbook (32). LVAD unloading generally relieves the load on the RV. However, preload can increase in specific conditions, such as RV dysfunction, TR, and LVAD-induced AI. Since claims especially regarding changes in preload after LVAD implantation vary depending on review papers (33, 34), we present actual hemodynamic data of ESP and EDP to support our discussion.

##### RV afterload

Since an LVAD relieves congestion on the left side of the heart and in the pulmonary circulation, RV afterload is generally expected to decrease following device placement. Below, we have listed three papers that report a reduction in RV afterload (ESP) or related components, including pulmonary capillary wedge pressure (PCWP) and mean PA pressure.

1. Data from Brener and colleagues demonstrated a slight but significant decrease in RV afterload during a temporary increase in LVAD speed (RV ESP 31.58 ± 9.75–29.58 ± 9.41 mmHg: *p* = 0.02) (35).
2. Regarding factors related to RV afterload, Masri and colleagues reported pulmonary artery catheter (PAC) data showing a significant reduction in PCWP (23.2 ± 7.6–14.9 ± 7.3 mmHg; *p* < 0.01) and mean PA pressure (from 35.9 ± 9.9 to 23.3 ± 7.7 mmHg; *p* < 0.01) within 72 h after LVAD implantation with a volume displacement pump (63%), axial pump (26%), and centrifugal pumps (11%) (36).
3. This trend generally continues over the long-term, as another study reported sustained decreases in PCWP (from 23 [1st and 3rd interquartile 17, 30] to 12 [7, 17] mmHg) and in effective arterial elastance (Ea; from 1.31 [0.7, 1.62] to 0.59 [0.42, 0.9] mmHg/ml) over six months after implantation of axial pumps (28%) and centrifugal pumps (72%) (37).

However, specifically in cases of AI, afterload may increase. Hemodynamic data comparing pressure parameters showed that patients who developed AI had higher mean PA pressure and PCWP compared to those without AI (38, 39).

##### RV preload

Preload, or RV end-diastolic pressure (EDP), decreases or remains stable immediately after LVAD implantation because of LVAD suction.

Below, we have listed three papers that report a reduction or stabilization in RV preload.

1. PV loop data from Brener and colleagues also demonstrated a slight decrease in preload (RV EDP 7.95 ± 3.55–7.42 ± 3.29 mmHg: *p* = 0.04).
2. Right atrial pressure (RAP) also generally decreases or stabilizes in the short term (RAP: 11.8 ± 6.5–10.1 ± 5.4 mmHg) after LVAD implantation with volume displacement pump, axial pump, and centrifugal pumps (36)
3. This trend generally continues over the long-term (RAP: 11 [1st and 3rd interquartile 5, 16], 10 [5, 15] mmHg) after implantation of axial pump and centrifugal pump (37).

Changes aforementioned are slight and biologically insignificant, suggesting that RV preload remains unchanged after LVAD implantation. However, in the presence of RV dysfunction, functional TR, or AI, preload may increase (40). The impact of AI and TR on the RV is discussed later in sections “3-1-4. Worsening of Functional Tricuspid Regurgitation” and “3-1-5. Aortic Insufficiency”.

Notably, there is an explanation suggesting that RV preload increases due to LVAD flow (34), probably based on data showing an increase in RV EDV (41). However, the studies examining changes in RV EDP, RAP, or CVP, not EDV, immediately after LVAD implantation or during ramp test, generally demonstrate either a decrease or stability in these values (35, 42, 43). An increase in RV EDV may result from improved RV compliance due to LVAD-induced suction; therefore, careful interpretation of these parameters is necessary to accurately assess true RV preload following LVAD implantation.

However, excessive LVAD speed remains a potential concern in LVAD management, because excessive LVAD speed may significantly impair RV contractility by disrupting LV twist and septal motion. In this dysfunctional RV, increased preload may be required to maintain stroke volume and circulatory equilibrium (41).

#### 3.1.4 Worsening of functional tricuspid regurgitation

##### - Increase in preload

Leftward shift of the septal wall can enlarge the tricuspid annulus, potentially inducing functional TR (44, 45). However, the real-world data has shown that over 50% of moderate-severe TR improves following LVAD implantation, attributed to the reduction in afterload (46–48). A particular proportion of patients (30%) still have persistent TR, and 6%–20% develops significant TR following surgery (46–48), both of which are associated with RHF development (44). Nakanishi and colleagues identified preprocedural tricuspid valve annular diameter as a predictor of persistent or worsening TR (49) and atrial fibrillation (AF) has also been reported as a potential predictor (48, 50). However, a large registry study from INTERMACS did not demonstrate a prognostic benefit of tricuspid valve procedures at the time of LVAD implantation, suggesting that the extent to which TR contributes to the development of right heart failure (RHF) remains unclear. Rather, TR may be a consequence of right heart enlargement secondary to RHF (51).

#### 3.1.5 Aortic insufficiency

##### - Increase in preload and afterload

AI increases preload on the LV and, ultimately RV (52). Indeed, AI has been linked to worsening mitral regurgitation following LVAD implantation, further supporting significant volume overload on the LV and subsequent negative effects on RV (53). Given its gradual progression, AI may contribute to late RHF.

Aortic insufficiency (AI) can develop over time due to LVAD outflow into the aorta. According to the STS INTERMACS registry, 15% of patients developed AI ≥moderate at 2 years after LVAD implantation, which was associated with poor outcomes (53). Importantly, this percentage gradually rose after LVAD implantation, having reached 37.6% within three years (54, 55).

At the time of LVAD implantation, it is reasonable to consider concomitant procedures on the aortic valve for more than mild AI and the outflow graft anastomosis should be oriented downstream to prevent the development of or progression of AI. However, outcome data on these interventions remain conflicting, and the effectiveness has not been clearly demonstrated (56). Following implantation, optimizing LVAD flow is also required to avoid excessive output to the aorta (54).

### 3.2 PV loop in patients with LVAD

This section summarizes data on the RV PV loop during a ramp test in patients with an LVAD, to capture true or intrinsic change of RV function due to LVAD suctioning.

Brener et al. published data in HeartMate3 recipients (*n* = 19) recruited from two sites (35). Based on the statistical analysis of ramp tests including all patients, they concluded the impact of an LVAD on the RV is as follows:

1. No significant decline in RV contractility
2. Improvement in RV diastolic compliance
3. Reduction in RV afterload

**Figure 4:**
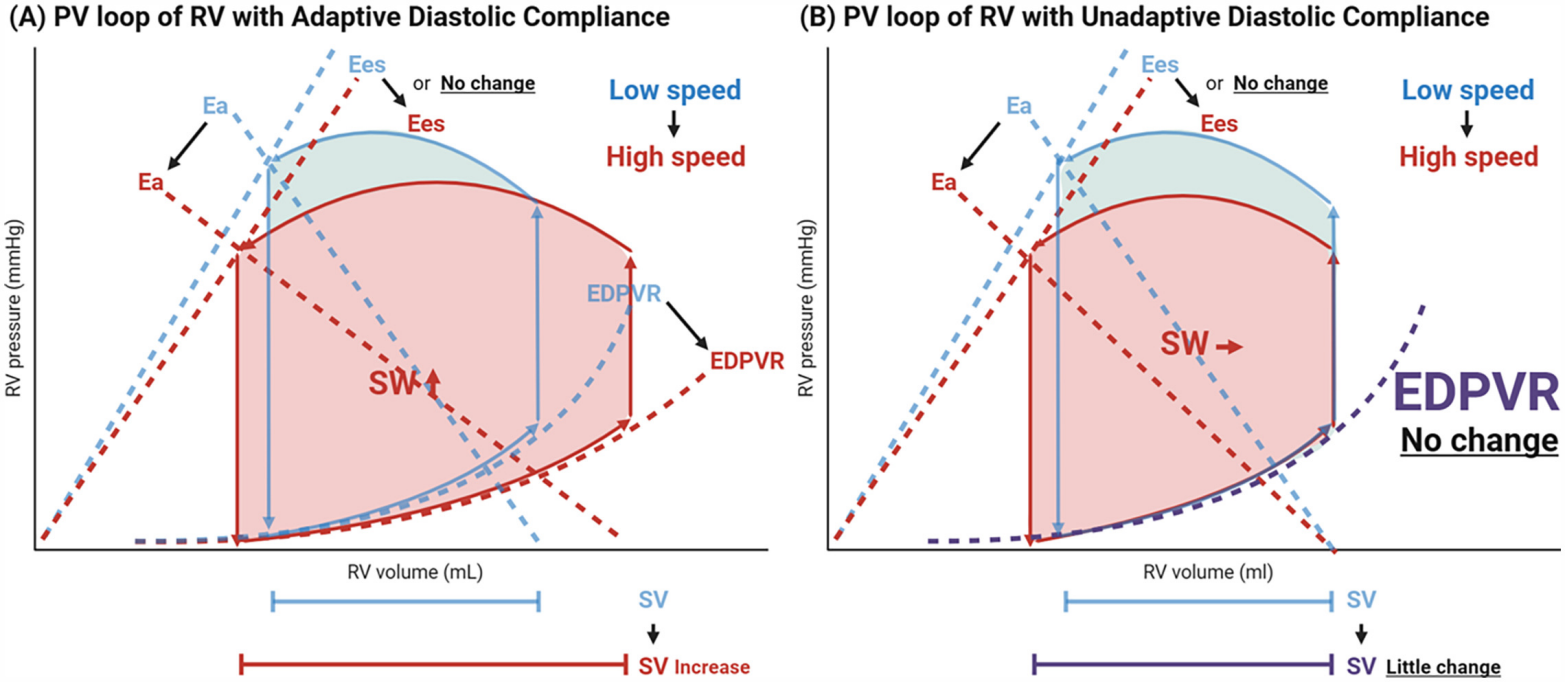
Transformation in right ventricular pressure-volume loop during ramp test. Depicted is the schematic illustration of RV PV loop transformation based on the current study (35). With the increase in LVAD pump speed, the EDPVR curve leans downwards, and the inclination of Ea line gets smaller (rightward/downward shift in RV PV loop), meaning that diastolic compliance improves, and afterload decreases **(A)** However, they identified certain recipients whose EDPVR does not improve and only RV afterload decreases (only downward shift) in response to pump speed, susceptive of inadaptive diastolic compliance, which may compromise hemodynamics and causes RHF **(B)** RV, right ventricle; PV, pressure-volume; Ea, effective arterial elastance; Ees, end-systolic elastance; EDPVR, end-diastolic pressure-volume relationship; SW, stroke work; SV, stroke volume; LVAD, left ventricular assist device; RVSWi, right ventricular stroke work index; RHF, right heart failure.

Notably, contractility did not significantly change, which contradicts the commonly held view that RV contractility declines due to loss of LV twist and impaired septum motion. In their study population, as pump speed was increased from 5,000 to 5,800 rpm, the RV PV loop expanded rightward and shifted downward, indicating an increase in SV and SW in response to higher pump speed. This change is attributed to “an improvement in diastolic compliance (EDPVR), also referred to as adaptive diastolic compliance in response to LVAD suctioning,” as well as “a reduction in afterload” (Figure 4A). From this perspective, therefore, the RV may accommodate the increased flow generated by the LVAD through enhanced diastolic function. In contrast, part of RVs in their study show inability to improve EDPVR, referred to inadaptive diastolic compliance, and only afterload is decreased in response to increased LVAD flow (Figure 4B), where PV loop shifts only downwards without rightward shift. These RVs failing to increase RV diastolic compliance, may be unable to generate a comparable SV, which may result in compromised hemodynamics and RHF.

Although the authors (35) concluded that contractility did not change, this interpretation should be treated with caution given the study limitation: (1) all patients were stable outpatients in a late postoperative phase, with a median of 144.5 days (interquartile range: 53.5–357 days) after LVAD implantation; (2) the small sample size (*n* = 19) limits the generalizability of the findings: and (3) only temporary changes were considered in this study, leaving uncertainty regarding the chronic impact of LVAD support. Further research with a larger, more heterogeneous cohort and longitudinal assessments is needed to draw more definitive conclusions about systolic function.

Besides, Scheel et al. also reported the measurement of RV Ees using conductance catheters in HeartMate 3 (Abbott, Chicago, IL) and HeartWare (Medtronic, Minneapolis, MN) recipients (*n* = 13) (30). During their ramp test (low, intermediate, and high speeds, increased by 100–200 rpm), RV Ees declined in specific cases. These findings differ from those reported by Brener et al., suggesting a heterogeneous impact on a case-by-case basis and underscoring that the negative effect of LVAD pump suction on RV contractility still cannot be overlooked.

Of note, all cases studied in the above reports were stable outpatients from 1 to 12 months after LVAD implantation, and thus their RVs may have been stable and managed well. Based on these results and previous data, we summarized the impact of LVAD on RV in the table on Figure 1.

### 3.3 RHF mechanism and knowledge gaps

Based on the latest evidence, we propose the following LVAD-specific mechanisms of RHF:

1. Inadequate Adaptation of Diastolic Function to Increased Flow: While diastolic compliance generally improves to accommodate the increased flow and generate more SV comparable to LVAD-generated SV, some RVs with unchanged diastolic compliance fail to provide the necessary SV, possibly leading to RHF (Figure 4B).
2. Reduced Contractility: RV contractility declines due to the loss of LV twist and reduced mobility of the septal wall caused by LVAD suction. Compromised contractility leads to RV-PA uncoupling and RHF, particularly in patients with high afterload, where the RV free wall fails to compensate for impaired septal movement.
3. Elevation of Preload: Dysfunctional RV and other factors (e.g., functional TR and AI) can increase preload chronically and result in RHF.

This represents the simplest scenario; however, other factors—such as the progression of underlying RV or pulmonary vessel diseases and *de novo* AF—can further compromise RV function and hemodynamic conditions. HF is invariably heterogenous.

The knowledge gap identified from the literature search is the scarcity of firm clinical evidence on proposed mechanisms, particularly on LV twist, and load-independent functional assessments using PV loop derived parameters such as Ees, EDPVR and Ees/Ea in real-world LVAD recipients. These limitations restrict the understanding of RHF within the expected range. Further data from human studies, including heterogenous patients are required to achieve a more definitive understanding.

## 4 Functional parameters and predictive value

Functional assessment of RV is essential to predict the likelihood of RHF following LVAD implantation (6). Severely depressed RV function prior to LVAD placement is one of the most significant predictors [odds ratio [OR] 1.60; 95% confidence interval [CI] 1.17–2.20; *p* = 0.004] in the multivariable analysis of STS–INTERMACS database (5–8). However, accurately assessing RV function remains a long-standing challenge. In end-stage HF, loading conditions fluctuate dramatically, undermining the reliability of functional parameters. To enhance the prediction of post-LVAD RHF, this section summarizes the relevant functional parameters, highlighting their utility and identifying the existing knowledge gaps.

The latest consensus statement recommends evaluating tricuspid annular plane systolic excursion (TAPSE), systolic tissue Doppler velocity of the tricuspid annulus (RV S'), right ventricular fractional area change (RV FAC), and right ventricular longitudinal strain (RV LS) prior to LVAD implantation (57). Echocardiography is the initial imaging modality of choice accessible for severely ill patients, with CT and MRI considered if echocardiographic findings remain inconclusive. Beyond these imaging modalities, right heart catheterization (RHC) parameters provide incremental value for assessing RV function in a load-independent manner, such as RVSWi, and pulmonary artery pulsatility index (PAPi) (58, 59). Although RAP/PCWP does not typically reflect RV function, it is included in this section as it is also derived from a RHC. This section highlights the predictive value (Tables 2–6), and limitations of these indices.

**Table 2:** Performance of systolic function parameters.

Study (population)	Performance	Cut off	Outcome	Comparison
TAPSE				
Raymer et al. (2019) (*n*=) (96)	AUC 0.67		RHF^a^	PAPi (AUC 0.63)
Patil et al. (2015) (*n* = 152) (97)	AUC 0.85	12.5 mm (Sens 84% Spec 75%)	RVAD implantation	
RV S’				
Kato et al. (2013) (*n* = 68) (67)	AUC 0.81	4.4 cm/s (Sens 87.5% Spec 68.2%)	RHF^a^	E/e’ (AUC 0.72) RV LS (AUC 0.75)
Dandel et al. (2010) (*n* = 205) (98)	AUC 0.90	8 cm/s (Sens 84% Spec 90%)	RHF^b^	
RV FAC				
Morita et al. (2018) (*n* = 80) (99)	AUC 0.80	15.9% (Sens 77.3% Spec 54.5%)	RVAD implantation	
Raina et al. (2013) (*n* = 55) (100)	AUC 0.67	31% (Sens 82% Spec 52%)		LAVI (AUC 0.71)
RV LS				
Liang et al. (2022) (*n* = 55) (101)	RVGLS OR 1.44	GLS −9.7% (Sens 0.89 Spec 0.78)	RHF^a^	FWLS (OR 1.23) TAPSE (OR 0.37) FAC (OR 0.91)
Dufendach et al. (2021) (*n* = 137) (102)	RVFWLS C-index 0.65 OR 1.14		1-year mortality	TAPSE (OR 0.44) PVR (OR 1.03)
Gumus et al. (2018) (*n* = 54) (77)	FWLS AUC 0.94	FWLS −15.5% (Sens 86.4% Spec 95.2%)	RHF^3^	RVSWI (AUC 0.82) FAC (AUC 0.72) RV EF (AUC 0.71)
Magunia et al. (2018) (*n* = 26) (103)	FWLS AUC 0.91		RVAD or inotropes >14 days	RVEF (AUC 0.88)
Comeli et al. (2013) (*n* = 10) (104)	FWLS AUC 0.93		RHF^a^	GLS (AUC 0.81) FAC (AUC 0.61) RV S’ (AUC 0.43) TAPSE (AUC 0.33)
Grant et al. (2012) (*n* = 177) (105)	FWLS AUC 0.70	FWLS −9.6% Sens 68%/Spec 76%	RVAD or Inotropes >14 days	RVFRS (AUC 0.66) (14)

TAPSE, tricuspid annular plane systolic excursion; AUC, area under the curve; RHF, right heart failure; PAPi, pulmonary artery pulsatility index; RVAD, right ventricular assist device; RV S’, right ventricular tissue Doppler S’ wave; RV LS, right ventricular longitudinal strain; FAC, fractional area change; FWLS, free wall longitudinal strain; RVGLS, right ventricular global longitudinal strain; GLS, global longitudinal strain; E/e’, ratio of early mitral inflow velocity to mitral annular early diastolic velocity; LAVI, left atrial volume index; RVEF, right ventricular ejection fraction; RVSWI, right ventricular stroke work index; PVR, pulmonary vascular resistance; RVFRS, University of Michigan right ventricular failure risk score.

^a^ Post-implant inotropic support >14 days, RVAD implantation for intra-operative or post-operative RHF, or death within 14 days due to RHF.

^b^ Need for the previously unplanned insertion of a RVAD after LVAD implantation or the necessity of both prolonged reduction of PVR by nitric oxide or iloprost inhalation and intravenous inotrope therapy for >10 consecutive days to increase the cardiac index >2 L/min per m^2^.

^c^ The mean arterial pressure <55 mmHg, central venous pressure or right atrial pressure >16 mmHg, cardiac index <2 L/min/m^2^, requirement of prolonged postimplant inotropes (inotropic score >20 units), or inhaled nitric oxide or intravenous vasodilators continued beyond postoperative day 14 following LVAD implant or requiring RVAD or extracorporeal membrane oxygenation support.

**Table 3:** Performance of diastolic function parameters.

Study (population)	Performance	Cut off	Outcome	Comparison
Trans tricuspid E/e’				
Kato et al. (2013) (*n* = 68) (67)	OR 1.32		RVAD or inotropes >14 days	TAPSE (OR 0.32) S’ (0.22) RV GLS (OR 1.26)
RA reservoir strain				
Charisopoulo et al. (2019) (*n* = 70) (68)	AUC 0.91 OR 2.5	10.5% (Sens 94% Spec 65%)	RVAD	FWLS (AUC 0.62; OR 1.3)
Catheter-derived RA waveform				
Samura et al. (2019) (*n* = 71) (69)	Deep Y descent OR 10.5		RVAD or inotropes >14 days	CVP/PCWP(OR2.02) PAPi (OR 1.13) RVSWi (OR 0.95)

RVAD, right ventricular assist device; TAPSE, tricuspid annular plane systolic excursion; S’, myocardial tissue Doppler systolic velocity; RV GLS, right ventricular global longitudinal strain; FWLS, free wall longitudinal strain; CVP, central venous pressure; PCWP, pulmonary capillary wedge pressure; PAPi, pulmonary artery pulsatility index; RWSWi, right ventricular stroke work index.

**Table 4:** Performance of RVSWi.

Study (population)	Performance	Cut off	Outcome	Comparison
RVSWI				
Gumus et al. (2019) (*n* = 57) (77)	AUC 0.82	400 mmHg^a^ml/m^2^	RHF^a^	FWLS (AUC 0.94) FAC (AUC 0.72) RV EF (AUC 0.71)
Bellavia et al. (2017) (*n* = 4428) (7)	SMD 0.58		Depends on the paper	CVP (SMD 0.47) TAPSE(SMD 0.29) FAC (SMD 0.29)
Kormos et al. (2010) (*n* = 484) (75)	OR 2.9	300 mmHg^a^ml/m^2^	RVAD or inotropes >14 days	RAP/PCWP(OR 2.5)

RWSWi, right ventricular stroke work index; AUC, area under the curve; SMD, standardized mean difference; RHF, right heart failure; FWLS, free wall longitudinal strain; FAC, fractional area change; RV EF, right ventricular ejection fraction; CVP, central venous pressure; TAPSE, tricuspid annular plane systolic excursion; RAP, right atrial pressure; PCWP, pulmonary capillary wedge pressure.

^a^ The mean arterial pressure <55 mmHg, CVP or RAP >16 mmHg, cardiac index <2 L/min/m^2^, requirement of prolonged postimplant inotropes (inotropic score >20 units), or inhaled nitric oxide or intravenous vasodilators continued beyond postoperative day 14 following LVAD implant or requiring RVAD or extracorporeal membrane oxygenation support.

**Table 5:** Performance of PAPi.

Study (population)	Performance	Cut off	Outcome	Comparison
PAPi				
Akamkam et al. (2024) (*n* = 170) (95)	AUC 0.68	2.84	3-month mortality	
Sheel et al. (2024) (*n* = 33) (106)	AUC 0.80		RV Ees/Ea <0.35	RVSWI (AUC 0.51) RAP/PCWP (AUC 0.52) CI (AUC 0.77)
Cacioli et al. (2022) (*n* = 54) (81)	Post NTP AUC 0.95	Post NTP PAPi 3.2	RHF^a^	CRITT score (AUC 0.72) EUROMACS (AUC 0.72)
Morine et al. (2016) (*n* = 132)	AUC 0.94	1.85	RVAD or inotropes >14 days	RAP/PCSP (AUC 0.84) RVSWI (AUC 0.69)

PAPi, pulmonary artery pulsatility index; AUC, area under the curve; RAP, right atrial pressure; PCWP, pulmonary capillary wedge pressure; CI, cardiac index; RWSWi, right ventricular stroke work index; NTP, normalized transpulmonary pressure; RHF, right heart failure; CRITT, cardiac risk, inflammatory response, timing of support, technical difficulty score; EUROMACS, European registry for patients with mechanical circulatory support; RVAD, right ventricular assist device; PCSP, pulmonary capillary systolic pressure; RV, right ventricular; Ees/Ea, end-systolic elastance to arterial elastance ratio.

^a^ Post-implant inotropic support >14 days, right ventricular assist device (RVAD) implantation for intra-operative or post-operative RV failure, or death within 14 days due to RV failure.

**Table 6:** Performance of RAP (CVP)/PCWP.

Study (population)	Performance	Cut off	Outcome	Comparison
RAP (CVP)/PCWP				
Beneyto et al. (2024) (*n* = 224) (73)	HR 1.35 AUC 0.62	0.33	6-month mortality	TR (HR 5.13) PVR (HR 1.11)
Mehra et al. (2022) (*n* = 1312) (93)	HR 1.57	0.60	1-year mortality	LVEDD <5.5 cm (HR 1.86)
Ruiz-Cano et al. (2020) (*n* = 80) (107)	OR 4.0	0.55	RHF^a^	TAPSE (*p* = 0.17) Severe TR (*p* = 0.34)
Akin et al. (2020) (*n* = 2,689) (108)	OR 1.46		90-day mortality	PAPi (OR 0.88; *p* < 0.001) RVSWI (OR 0.91; *p* < 0.001) TAPSE (OR 0.99; *p* = 0.48)
Samura et al. (2019) (*n* = 115) (88)	OR 2.02		RVAD or inotropes >14 days	RVSWI (OR 0.95) PAPi (OR 1.13)

RAP, right atrial pressure; CVP, central venous pressure; PCWP, pulmonary capillary wedge pressure; HR, hazard ratio; OR, odds ratio; AUC, area under the curve; RHF, right heart failure; RVAD, right ventricular assist device; TR, tricuspid regurgitation; PVR, pulmonary valve resistance; TAPSE, tricuspid annular plane systolic excursion; PASP, pulmonary artery systolic pressure; PAPi, pulmonary artery pulsatility index; LVEDD, left ventricular end-diastolic dimension; RWSWi, right ventricular stroke work index.

^a^ Evidence of CVP >16 mm Hg with a CI <2.3 L min^−1^m^−2^ (in the absence of elevated PCWP, tamponade, ventricular arrhythmias or pneumothorax) after LVAD implantation requiring previously unplanned temporary RVAD) or the necessity of nitric oxide (iNO) and intravenous inotropes beyond postoperative day 14.

### 4.1 Systolic function

TAPSE, RV S', RV FAC, and RV LS are the most commonly used parameters for measuring RV systolic function. A meta-analysis demonstrated that TAPSE, RV FAC, and RV global longitudinal strain (GLS) reliably distinguish between patients who do and do not develop RHF (60). Among these parameters, RV free-wall longitudinal strain (FWLS) is specifically recommended for evaluating subclinical RV dysfunction in LVAD candidates (57). Indeed, RV LS generally demonstrates superior performance in predicting RHF and adverse events, in comparison to TAPSE, RV FAC (Table 2).

***Limitations:*** However, it is important to note that these parameters represent geographic or volumetric changes in the RV, rather than “contractility” or “Ees” derived from the PV loop, strictly speaking (61). Consequently, they have load-dependency, which is a critical limitation in capturing intrinsic RV function data in severe heart failure, where afterload and preload can dramatically fluctuate (10–12). Although Ees derived from the PV loop is considered the gold standard for assessing contractility independently of loading conditions, its routine use in daily clinical practice is limited by the invasiveness, analytical complexity, and high cost (62).

Additionally, all these assess only regional function, and TAPSE and RV S' have angle dependency, thus requiring clinicians to be cautious when interpreting actual values (61). Given the complex structure of the RV, three-dimensional ejection fraction would be ideal to capture more global motion (63), yet this approach is technically challenging to accurately visualize and is not widely available in real-world clinical settings, as indicated in current guidelines (64).

Moreover, the median values of TAPSE (1.03–1.61 cm), RV FAC (18.7%–40.4%), and RV GLS (6.7%–12.6%) in patients who developed RHF vary widely across studies (60), suggesting their unreliability as definitive predictors of RHF. Therefore, careful consideration of simultaneous loading conditions, or other load-dependent indices is highly recommended.

### 4.2 Diastolic function

Given the discussion in the previous section of pathophysiology, the adaptability of diastolic function can be a key contributor to post-LVAD RHF development, yet the data remains particularly scarce (65). In echocardiography or MRI, trans-tricuspid E/A ratio, deceleration time of peak E velocity, E/e', RV diastolic strain rate (DSR), or right atrial (RA) strain are used to assess RV diastolic function (66). E/e' is associated with RV filling pressure, and E/e' > 10 may be helpful in predicting post-LVAD RHF. However, the supporting evidence is limited to univariable analysis, and further investigation is warranted (67). In addition, impaired peak RA strain, which also reflects RV filling pressure or RA reservoir function, was reported as another independent predictor of subsequent RVAD implantation (68). Their analysis demonstrated excellent predictive values of diastolic functional parameters including peak RA strain and late diastolic strain rate, outperforming RV LS.

Furthermore, catheter-derived RA waveforms show better predictive values for RHF. A deeper Y descent compared to X descent, indicative of impaired RV diastolic compliance, demonstrated a high OR of 10.5 (95% CI 1.75–63.5), surpassing other catheter-derived parameters such as central venous pressure (CVP)/PCWP, PAPi, and RVSWi (69).

***Limitations:*** As a significant knowledge gap, evidence pertaining to diastolic function remains extremely limited, and even studies investigating systolic function often do not compare their findings to diastolic function parameters (Table 3). However, the data on diastolic function parameters identified thus far have demonstrated excellent utility in predicting post-LVAD RHF or mortality, comparable or even superior to RV LS, and further studies are strongly warranted.

### 4.3 RV-PA coupling

RV Ees/Ea, or RV-PA coupling, serves as a mediator of RV contractility while accounting for PA elastance or afterload, and is primarily measured using a conductance catheter and PV loop. Scheel and colleagues demonstrated that a lower RV Ees/Ea (below 0.35) was associated with more frequent heart failure symptoms compared to a RV Ees/Ea ≥0.35 (71% vs. 17%, *p* = 0.048), suggesting that this parameter effectively captures an uncoupled, abnormal RV state by incorporating loading conditions (30, 42). However, as previously mentioned, data obtained using conductance catheters and PV loops remains limited.

Recently, the ratio of echocardiographic systolic parameters, such as TAPSE, RV S', RV LS, and approximated pulmonary artery systolic pressure (PASP), have been employed as a surrogate of RV Ees/Ea (61). This echocardiographic surrogate has been adopted across various patient populations (70), yet the utility in LVAD population is unclear, with studies indicating that this parameter lacks predictive value for RHF development (71–73).

***Limitations:*** A few limitations should be considered: (1) PASP estimation is challenging in cases of severe or greater TR due to the widened regurgitant orifice, which hinders the accurate capture of the pressure gradient; (2) TAPSE, RV S', and RV LS do not fully capture global RV systolic function, as they provide only regional assessments, which is problematic in an enlarged, dysfunctional RV; (3) in end-stage RV dysfunction, PASP may not increase as expected due to the RV's inability to generate sufficient flow and pressure, being critical to use PASP as a surrogate of Ea (66, 70). Although RV-PA coupling is reliable indices, adoption of echocardiography estimate in LVAD is not clearly explored.

### 4.4 RVSWi

The right ventricular stroke work index (RVSWi) reflects the RV external workload, representing the area enclosed by the PV loop. It is obtained from PV loops using a conductance catheter or estimated using RHC-derived parameters based on the following formula:

RVSWI=(meanPApressurendashCVP)×SVindex(×0.0136)

RVSWi is considered a load-independent parameter and is particularly useful in evaluating complex pathophysiology, where loading conditions are highly variable (74). In general, RVSWi decreases in dysfunctional RV because low Ees and impaired EDPVR are unable to generate sufficient external workload (74). Specifically, RVSWi <250, 300, or 400 mmHg L/m^2^ are used for cut-off points for predicting post-LVAD RHF (75–77).

The recent meta-analysis demonstrated the highest standard mean difference of RVSWI (0.58) among various RV hemodynamic and functional parameters such as TAPSE, RV FAC, and RV LS, although PAPi and RAP/PCWP were not included in this analysis (7).

***Limitations:*** RVWSi is frequently used in LVAD populations (Table 4), yet most RVSWi measurements used in clinical, or research settings are derived from RHC-derived parameters. These estimations do not necessarily capture true workload as measured from PV loops and are susceptible to errors in hemodynamic measurements (77). Moreover, there are also some studies that have shown limited predictive capacity of RVSWi in multivariable analyses (78). The specific situation or population where its performance becomes unreliable is still unclear, it should be noted that functional assessment should be multifaceted.

### 4.5 PAPi

The pulmonary artery pulsatility index (PAPi) is a measure initially developed for use in MI and MCS conditions and it has demonstrated a good predictive value for RHF development or mortality after LVAD implantation either (79). PAPi is calculated using the following formula:

PAPi=(systolic-diastolicPApressure)/RApressure,

where PA pulse pressure provides an estimate of RV pulsatile load and contractile strength, and RA pressure acts as a mediator of preload (80). Lower PAPi indicates impaired RV function. A systematic review incorporating 32 studies found that patients who developed RHF had a significantly lower preoperative PAPi than those who did not (2.17 vs. 2.87; *p* < 0.001) (79) (Table 5).

In analysis on PAPi, the utility of simulation test has been proposed in addition to the resting value. Cacioli and colleagues reported PAPi measured after administering sodium nitroprusside (NTP) had the potential to predict RHF development. By administrating the vasodilator and reducing pulmonary vascular resistance, post-NTP PAPi was considered capable of assessing (1) reversibility of pulmonary hypertension and (2) the reserve in RV function. Indeed, the post-NTP PAPi improved predictive value, and when combined with RV FAC and systolic PA pressure, the area under the curve (AUC) increased up to 0.95 in their study (81). Hsi and colleagues also reported the efficacy of PAPi measured in a micro-axial flow pump (mAFP) test to predict RHF before LVAD implantation (82). These dynamic changes in parameters during simulation testing may help capture the intrinsic adaptability of the RV and improve the prediction of RHF (83). Although this was also suggested in the study by Brener and colleagues where they proposed RVSWi (35), available data remains limited.

***Limitations:*** A concern regarding PAPi is that pulmonary circulation factors—including resistance and compliance—can significantly affect its value. Not only RV dysfunction but also pulmonary embolism and PA diseases can diminish PAPi value since pulse pressure declines in these conditions (84).

Another concern is the variable cutoff value reported in literature. From 0.88 to 3.3 have been reported as an effective cutoffs, and median values also vary across studies (85). Even small changes in RA pressure can significantly affect PAPi, precise measurement of catheter parameters is essential.

### 4.6 RAP (CVP)/PCWP

RAP (or CVP)/PCWP includes the LV component when evaluating the RV preload, thereby reflecting right-sided preload relative to the left side and implying RV's adaptability towards certain volume condition, not representing RV function (86). With respect to prediction, RAP/PCWP ratio shows excellent predictive values (87, 88). A higher RAP/PCWP ratio indicates excessively accumulated preload in the RV, suggesting inadequate adaptation of RV to certain volume conditions or afterloads. In general, 0.55–0.6 are considered as cutoff values (58) (Table 6).

Regarding this parameter, data are available not only for its resting value at a single time point but also from a simulation test of LVAD suctioning. In terms of this parameter, there is a data of utility by simulation test of LVAD, not only rest value at one point. Hsi and colleagues reported the efficacy of a simulation test with mAFP in assessing the RAP/PCWP ratio either (82). In their study, the ratio decreased significantly after mAFP insertion in patients who did not develop RHF, compared to those who did (non-RHF group: 0.61 → 0.38; RHF group: 0.46 → 0.40) (82). Simulation test performed before implantation may further improve the predictive value of this parameter as PAPi. RAP/PCWP is simple to obtain and has a high predictive reliability; therefore, its assessment is reasonable and recommended.

***Limitations:*** As a limitation, this ratio can be low when PCWP is particularly high. Amsallem and colleagues reported that 43% of patients with a low RAP/PAWP ratio (≤0.54) and high RAP (≥15 mmHg) developed RHF after LVAD implantation, suggesting that the absolute value of RAP itself should also be considered (89).

## 5 Risk sores and significant items for prediction

Various scoring models, many of which do not focus solely on RV factors, have been developed to predict the incidence of post-LVAD RHF. Although incorporating the non-RV-centered factors helps further identify high-risk patients for developing RHF prior to LVAD implantation, comprehensive validation of these models and comparisons of their performance remains limited.

This section outlines several risk scores validated externally (Table 7). Components of these scores can be classified as follows: (1) baseline characteristics/presentation; (2) treatment-related factors (e.g., vasopressors, inotropes, MCS); (3) right heart conditions (hemodynamics, and RV function); (4) laboratory data. Most scores define RHF as the need for MCS or RVAD, iNO ≥48h, or inotropes ≥14 days, same as MCS-ARC early RHF definition (14).

**Table 7:** Summary of existing risk scores.

Risk score	Included Risk factors (weight)	Performance	Definition of RHF	Performance in validation (comparison)
STOP-RHF Score (*n* = 798) Taleb et al. (2024) (87)	Baseline background/presentation: • NICM (Yes/No) • INTERMACS 1–2 (Yes/No) Therapy: • IABP (Yes/NO) • Impella/VA-ECMO (Yes/No) • LVAD configuration (Centrifugal/axial) • ACEi (Yes/No) Right heart: • RAP/PCWP (value) Laboratory data: • Albumin (value) • Creatinine (value) • Platelet (value) Serum sodium (value)	C-index 0.75 AUC 0.75	1) and/or 2) within 30 days 1. Right-side circulatory support 2. inotropes therapy for ≥14 days	1) C-index 0.73 (University of Utah: C-index 0.62) (87)
EUROMACS-RHF (*n* = 2,000) Soliman et al. (2018) (88)	Baseline background/presentation: • INTERMACS class 1–3 (2 points) • ≥3 intravenous inotropes (2.5 points) Right heart: • Severe RV dysfunction (2 points) • RAP/PCWP > 0.54 (2 points) Laboratory data: haemoglobin ≤10 g/dl (1 point)	C-index 0.70 (cut off 2.5 pts Sens 74%/Spec 57%)	At least one of three 1. need for right side MCS 2. inotropic support for ≥14 days iNO for ≥48 h	1) C-index 0.67 (88) 2) AUC 0.64 (CRIIT: AUC 0.64/ University of Utah: AUC 0.57/ RVFRS: AUC 0.58) (109) 3) AUC 0.67 (110) 4) AUC 0.59 (111)
CRIIT score (*n* = 218) Alturi et al. (2013) (94)	Baseline background/presentation: • Pre-operative intubation (1 point) • Tachycardia >100 (1 point) Right heart: • Severe RV dysfunction (1 point) • CVP >15 mmHg (1 point) Severe tricuspid regurgitation (1 point)	C-index 0.8 AUC 0.80 (95% CI 0.72–0.88) sens 84%/spec 63% (cut off 2 pts)	Need for biventricular support	1) C-index 0.60 (University of Utah: C-index 0.59/ University of Pennsylvania: C-index 0.56) (90) 2) AUC 0.64 (109)
University of Utah (*n* = 175) Drakos et al. (2010) (91)	Baseline background/presentation: • Destination therapy (3.5 points) • Obesity (2 points) Therapy: ➢ IABP (4 points) ➢ Inotrope dependency (2.5 points) • ACEi/RAS (2.5 points) • β blocker (2 points) Right heart: Increased PVR (1–4 points)	Points and RHF (%) ≤5.0 (11%) 5.5–8 (37%) 8.5–12 (56%) ≥12.5 (83%)	At least one of the three 1. iNO for ≥48 h 2. Iinotropes for >14 days RVAD implantation	1) C-index 0.59 (90) 2) AUC 0.57 (109) 3) C-index 0.62 (87)
RVFRS (*n* = 197) Matthews et al. (2008) (112)	Therapy: • Vasopressor (4 points) Laboratory data: • AST ≥80 IU/L (2 points) • Bilirubin ≥2.0 mg/dl (2.5 points) Creatinine ≥2.3 mg/dl (3 points)	AUC 0.73	At least one of the four 1. Inotropes for >14 days; 2. iNO for ≥48 h; 3. right-sided circulatory support 4. hospital discharge with inotrope	1) C-index 0.62 (90) 2) AUC 0.61 (90) 3) AUC 0.58 (109)
University of Pennsylvania (*n* = 266) Fitzpatrick et al. (2008) (76)	Baseline background/presentation: • SBP ≤96 mm Hg (13 points) • Cardiac index ≤2.2 L/min/m^2^ (18 points) Right heart: • RVSWI ≤0.25 mm Hg L/m^2^ (18 points) • Severe pre-VAD RV dysfunction (16 points) Laboratory data: • Creatinine ≥1.9 mg/dl (17 points)	Sens 83%/spec80% (cut off 50 pts)	Need for MCS	C-index 0.56 (90)

Discrimination performance generally ranges from “fair (AUC 0.5–0.7)” to “acceptable (AUC 0.7–0.8)”. Of them, liver/renal dysfunction, MCS/catecholamine dependency, and high RAP/PCWP are factors used in at least two scoring models and thus should be incorporated certainly to evaluate the risk of RV development.

AST, aspartate aminotransferase; AUC, area under curve; BiVAD, biventricular assist device; CVP, central venous pressure; IABP, intra-aortic ballon pumping; INR, international normalised ratio; INTERMACS, Interagency Registry for Mechanically Assisted Circulatory Support; LVAD, left ventricular assist device; NICM, non-ischaemic cardiomyopathy; RA/PCWP, right atrium to pulmonary capillary wedge pressure ratio; RHF, right heart failure; RV, right ventricle; RVSWI, right ventricular stroke work index; SBP, systolic blood pressure; VAD, ventricular assist device.

**Figure 5:**
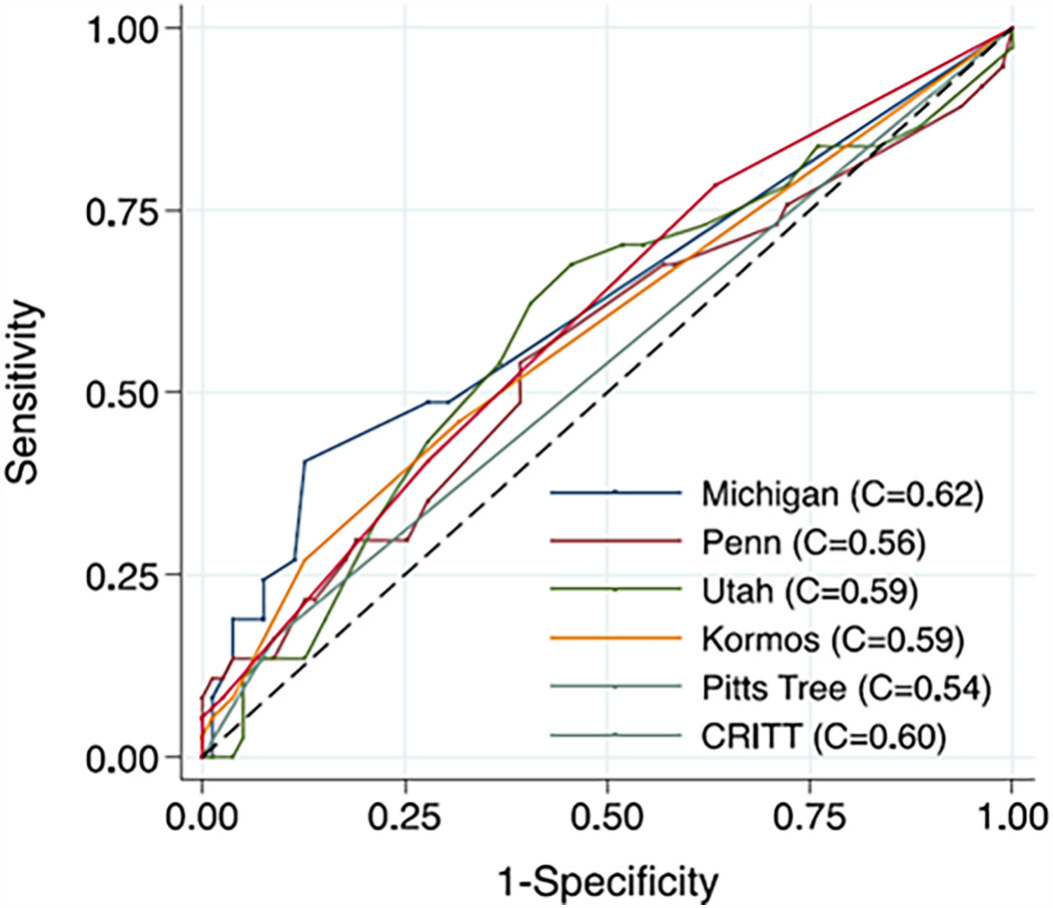
Comparison of discrimination of risk scores. Kalogeropoulos et al. compared existing risk scores in 116 patients with continuous-flow LVAD, either the HeartMate II (Thoratec, Inc.) or the HeartWare HVAD (Heart-Ware, Inc.), in single center. Although the Michigan score showed the highest value, the C-indexes of all scores remained around 0.6. Reproduced with permission from “Comparative Receiver Operating Characteristic Curve Plots with Right Ventricular Failure Prediction as the Outcome of Interest for the Scores Evaluated” by Andreas P. Kalogeropoulos, Anita Kelkar, Jeremy F. Weinberger, Alanna A. Morris, Vasiliki V. Georgiopoulou, David W. Markham, Javed Butler, J. David Vega and Andrew L. Smith, licensed under CC-BY-NC-ND.

As summarized in Table 7, the discrimination performances of most scores are generally “fair” or “moderate,” with C-index or AUC values less than 0.7, and comparable results observed in external validations. Scores relatively recently developed, including STOP-RVF Score, EUROMACS Score, and CRITT, are derived from cohort all with continuous flow pump, whereas that for other scores included pulsatile flow pump. Kalogeropoulos and colleagues compared six existing risk scores in their cohort who were all recipients of continuous flow LVAD, with the Michigan Right Ventricular Failure Risk Score (RVFRS) achieving the highest C-index of 0.62 (Figure 5). They attribute the limited performance to: (1) Variability in RHF definitions, (2) The inclusion of non-RV markers as surrogates for RV dysfunction, and (3) Differences in the cohorts used for model derivation, as older scoring systems included fewer patients receiving destination therapy and continuous-flow pumps (90).

The most recent risk prediction model, called “STOP-RVF score”, derived using a machine learning approach, has demonstrated superior performance, achieving a C-statistic of 0.75 even in external validation, outperforming other scores such as the Kormos et al. score [C statistic, 0.5 (75)] and score from the University of Utah [C statistic, 0.627 (91)] (87).

Noticeably, four items—(1) liver/renal dysfunction, (2) MCS/catecholamine dependency (or INTERMACS profile 1/2) before implantation, (3) pre-existing severe RV dysfunction, and (4) high RAP/PCWP—are included in at least two scoring models and thus, should be incorporated certainly when evaluating the RHF risk (Figure 1). Meta-analysis or large-scale registry studies (*n* > 1,000) conducting multivariable analysis for the incidence of post-LVAD RHF, also demonstrated the statistical significance of the aforementioned four factors (7, 8, 92, 93).

Unfortunately, “severe RV dysfunction” is not clearly defined in most studies including risk score deviation paper (76, 88, 94), yet, given their superior predictive capacities discussed in previous section, RV LS, RVSWi, and PAPi should be evaluated. Considering preimplantation RV LS ≥16% (77), RVSWi <250–300 mmHg L/m^2^ (75–77), or PAPi <2.0–3.0 (95) as a critical factor strongly indicative of RV dysfunction, patients with additional risk factors—such as liver/renal dysfunction, MCS/catecholamine dependency, or high RAP/PCWP (0.5–0.6)—should be strongly considered for RVAD implantation.

## 6 Conclusion

This review consolidates the latest insights into the pathophysiology of and predictive models for RHF following LVAD implementation.

As highlighted in Figure 1, both the loss of LV twist and LVAD suction on the septal wall are LVAD-specific factors that negatively impact RV contractility. Although LVAD suction improves diastolic compliance, insufficient adaptation to increased flow may contribute to RHF. Furthermore, functional TR and AI can additionally promote congestion.

To predict this unfavorable outcome, guidelines and expert consensus recommend several RV functional parameters from imaging modalities or RHC. Among these, RV LS, RVSWi, and PAPi, exhibit strong predictive value in large-scale studies. Since none of these parameters alone provide definitively high predictive accuracy, and each reflects different aspects of RV function, considering multiple parameters in combination may enhance the prediction of RHF.

Although several risk scores have been developed for prediction, most have demonstrated only “fair” performance in discriminating RHF during external validation. Across these score models, four specific factors recur and have demonstrated consistent performance in meta-analysis either, thereby we propose multiparametric approach for RHF prediction, specifically focusing on four factors—(1) liver/renal dysfunction, (2) MCS/catecholamine dependency (or INTERMACS profile 1/2), (3) pre-existing severe RV dysfunction (impairment in RV LS, RVSWi, and PAPi), and (4) high RAP/PCWP (Figure 1).

We identified the following knowledge gaps and future directions:

1. Incomplete Clinical Evidence on RV Pathophysiology in LVAD Candidates

The proposed pathophysiological mechanisms are largely derived from preclinical studies or estimations based on imaging modalities or RHC, where contractility and diastolic compliance cannot be accurately assessed. More robust evidence on proposed mechanisms, particularly the loss of LV twist, along with comprehensive, larger-scale evaluations of the RV PV loop in human patients, is needed.

2. Evaluation of Diastolic Compliance

Although diastolic compliance may significantly influence how the RV adapts to the increased flow from an LVAD, standard assessment methods via imaging modality or RHC for diastolic function remain limited. Further research is required to collect data on current diastolic function metrics and develop reliable parameters.

3. Simulation and Intraoperative Testing

Simulation studies that replicate LVAD hemodynamics, as well as short-term intraoperative tests just before LVAD implantation, could enhance predictive accuracy by providing more precise assessments of RV functional reserve. However, comprehensive and refined data are still needed.

Addressing these gaps is crucial for improving LVAD management and enhancing quality of life for patients reliant on LVAD support.
